# Difference in left atrial appendage remodeling between diabetic and nondiabetic patients with atrial fibrillation

**DOI:** 10.1002/clc.23292

**Published:** 2019-11-22

**Authors:** Chaim Yosefy, Marina Pery, Roman Nevzorov, Xavier Piltz, Azriel Osherov, Jamal Jafari, Ronen Beeri, Enrique Gallego‐Colon, Aner Daum, Vladimir Khalameizer

**Affiliations:** ^1^ Cardiology Department Barzilai University Medical Center, Ben‐Gurion University Ashkelon Israel; ^2^ Diagnostic Cardiology Unit Heart Institute, Hadassah Hebrew University Medical Center Jerusalem Israel

**Keywords:** atrial fibrillation, diabetes mellitus, left atrial appendage, left atrium, real‐time 3‐dimensional transesophageal echocardiography, stroke

## Abstract

**Background:**

Diabetes mellitus (DM) is a common and increasingly prevalent condition in patients with atrial fibrillation (AFib). The left atrium appendage (LAA), a small outpouch from the LA, is the most common location for thrombus formation in patients with AFib.

**Hypothesis:**

In this study, we examined LAA remodeling differences between diabetic and nondiabetic patients with AFib.

**Methods:**

This retrospective study analyzed data from 242 subjects subdivided into two subgroups of 122 with DM (diabetic group) and 120 without DM (nondiabetic group). The study group underwent real‐time 3‐dimensional transesophageal echocardiography (RT3DTEE) for AFib ablation, cardioversion, or LAA device closure. The LAA dimensions were measured using the “Yosefy rotational 3DTEE method.”

**Results:**

The RT3DTEE analysis revealed that diabetic patients display larger LAA diameters, D1‐lengh (2.09 ± 0.50 vs 1.88 ± 0.54 cm, *P =* .003), D2‐width (1.70 ± 0.48 vs 1.55 ± 0.55 cm, *P =* .024), D3‐depth (2.21 ± 0.75 vs 1.99 ± 0.65 cm, *P* = .017), larger orifice areas (2.8 ± 1.35 and 2.3 ± 1.49 cm^2^, *P* = .004), and diminished orifice flow velocity (37.3 ± 17.6 and 43.7 ± 19.5 cm/sec, *P* = .008).

**Conclusions:**

Adverse LAA remodeling in DM patients with AFib is characterized by significantly LAA orifice enlargement and reduced orifice flow velocity. Analysis of LAA geometry and hemodynamics may have clinical implications in thrombotic risk assessment and treatment of DM patients with AFib.

## 1 INTRODUCTION

Chronic hyperglycemia and its associated long‐term complications are one of the most significant causes of morbidity and mortality worldwide.^1^ The severity and duration of the diabetic state is considered a strong predictor in the development of macro‐ and microvascular complications including intima‐media thickness, coronary vascular disease (CVD), and stroke.^2^, ^3^ In fact, at least 68% of diabetes mellitus (DM) patients older than 65 years old will die of a CVD‐associated condition.^3^, ^4^ DM can cause heart failure due to poor left ventricular tolerance to ischemia and electrical disturbances.^1^, ^5^

Studies have shown an increased incidence of AFib in diabetic patients.^6^, ^7^ In a meta‐analysis including 108 703 patients with atrial fibrillation (AFib) from seven prospective cohort and four case‐control studies, DM was independently associated with an overall 40% increase in the risk of AFib.^7^ Several factors have been suggested to contribute to the development of AFib in DM patients including, hyperglycemia, insulin resistance, inflammatory milieu, and endothelial dysfunction.^8^ All these factors promote structural, electrical, electromechanical, and autonomic left atrium (LA) remodeling resulting in abnormal blood flow and stasis, which can potentially increase the risk for thrombus formation and stroke.^9^ In the clinics, the CHA_2_DS_2_‐VASc score, which includes DM, estimates the risk of stroke in patients with AFib. The left atrial appendage (LAA), a complex, small pouchlike sac protruding from the LA, is the main site for thrombus formation in patients with AFib.^10^ Altered LAA geometry increases the probability of thromboembolic event notwithstanding lower CHA_2_DS_2_‐VASc scores.^11^, ^12^ In addition to increase risk of stroke, structural and functional remodeling of LAA is related to the genesis and perpetuation of chronic AFib.^13^, ^14^, ^15^, ^16^, ^17^, ^18^ The purpose of the present study was to evaluate changes in LAA geometry, architecture, and function in patients with AFib, with and without DM.

## 2 MATERIALS AND METHODS

### 2.1 Study population

For this retrospective cohort study, we analyzed a matched group of patients with persistent AFib with and without DM, at our hospital from January 2014 to March 2018. Persistent AFib was defined as AFib that lasts longer than 7 days, including episodes that are terminated by cardioversion, either with drugs or by direct current cardioversion, after 7 days or more.^19^ The study population included 242 patients with AFib (>18 months) divided into two subgroups of 122 with DM (diabetic group) and 120 without DM (nondiabetic group) undergoing transesophageal echocardiography (TEE). Patients in the DM presented with an HB1Ac of 8.3% in average. The exclusion criteria included (a) patients ≤18 years old and (b) patients with significant valvular disease, mechanical or infected valves according to the American Society of Echocardiography Guidelines.^20^ All subjects underwent 2D TTE and real‐time 3‐dimensional transesophageal echocardiography (RT3DTEE) with full echocardiographic measurements before AFib ablation, cardioversion, or LAA device closure according to current guidelines.^20^, ^21^ All clinical and demographic characteristics were collected from hospital medical records and correlated with echocardiographic findings for LAA geometry. The study protocol adhered to the Declaration of Helsinki and was approved by the institutional review board.

### 2.2 Two‐dimensional TTE

All routine echocardiography exams used EPIQ 7 and iE33 echo machine (Philips Medical Systems, Andover, MA). All images were digitally stored for offline analysis (QLAB 10.0 cardiac 3DQ, Philips Medical Systems). All echocardiographic measurements for cardiac chambers were done according to the guidelines^20^, ^21^ and normalized for the body surface area. We assessed the length, width, depth, area, orifice flow velocity, spontaneous echo contrast (SEC), presence of thrombi, and lobe number of the LAA in patients with AFib, with and without DM.

### 2.3 Yosefy rotational 3‐dimensional TEE method

**Figure 1:**
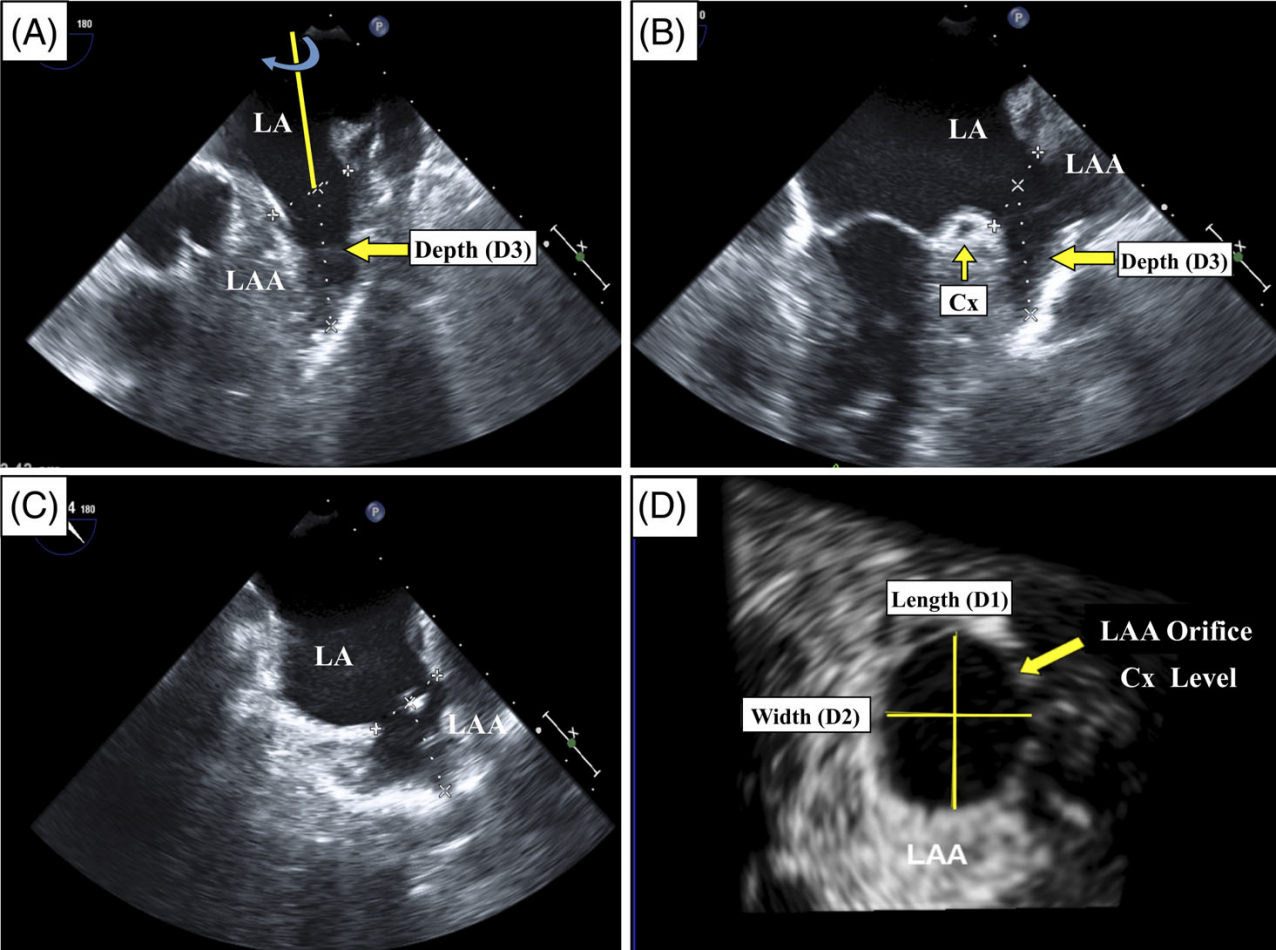
3D TEE analysis of the LAA maximal parameters measurement, at “one stop shop” point, using 360° “Yosefy rotational 3DTEE method”. A, After exclusion of thrombi and verifying the lobe structure with the rotation, measurement of depth (D3) can be made directly at this point. B, The maximal and minimal LAA diameters (D1‐length and D2‐width, respectively) at the level of the Cx artery can be measured more accurately on the orthogonal view (C). D, 3D imaging of “Yosefy Rotational 3DTEE method”. CX, circumflex; LA, left atrium; LAA, left atrium appendage

The 3D protocol for LAA dimension measurements and data acquisition includes the entire LAA in 3D Zoom mode.^22^ Using the 3DQ application on QLab, the ECG guided end systole (ie, end of T‐wave) is located for adequate measurement of maximal LAA dimensions. Magnified multiplanar reconstruction 2D images were adjusted, and the three axis lines (X, Y, and Z) were cropped to optimal alignment. After optimizing the blue line to the circumflex artery level and decreasing the gain in the volume mode, the sagittal plane is selected and screened in a 360° rotation view (Figure 1 and Figure S1). This 360° rotation or “Yosefy rotational 3DTEE method” is a simpler and faster 3D approach that provides all the LAA parameters needed in a single “one stop shop” image with adequate intra‐ and interobserver variability (*r* = 0.97, 0.95, 0.95, and 0.97 and *r* = 0.95, 0.94, 0.99, and 0.95, respectively).^22^ The image includes the number of lobes (during the rotation), the orifice area, the maximal and minimal diameters (D1‐length and D2‐width, respectively) and depth (D3‐depth) (Figure 1). The LAA measurements are equivalent to the accuracy of cardiac CT.^23^ This method is routinely used at our hospital for LAA closure procedures involving thrombi exclusion, determination of proper device size and implantation.

### 2.4 Statistical analysis

The results are presented as the mean ± SD for continuous variables with normal distribution, as the interquartile range for continuous variables with abnormal distribution, and as number and percentage of total patients for categorical data. *t* test was used for comparison of continuous variables. When the distribution was abnormal, the Mann‐Whitney *U* test was applied accordingly. *χ* ^2^ test and Fisher's exact test were used for categorical data. A two‐sided *P*‐value <0.05 was considered statistically significant. Logistic regression analysis was used to determine the variables associated with LAA geometry and to calculate the adjusted odds ratios (ORs) for significant comorbidities and echocardiographic measurements. Velocities >40 cm/s are suggestive of adequate flow within the LAA and a low risk for thrombus formation.^24^ For multivariate analysis, LAA flow velocity ≤40 cm/s was considered reduced. The initial selection of the variables in the model was based on univariate analysis with inclusion criteria of *P* < .05. The results are presented as the OR with 95% confidence interval (CI). Statistical analysis was performed with SPSS software version 21.0 statistical package (SPSS IBM Inc.).

## 3 RESULTS

### 3.1 Baseline characteristics

Baseline patient characteristics are shown in Table 1. Based on clinical indications, TEE was performed in 242 patients with AFib. The DM group, comprising 122 patients, was compared with 120 patients from the non‐DM group. There were no significant differences in age, sex, dyslipidemia, ischemic heart disease, chronic heart failure, peripheral vascular disease, stroke, BMI, BSA, hypertension, and systolic and diastolic blood pressure between the two groups. Hypertension was predominantly observed in both groups (83.6% vs 80.1%; *P* = .07). The CHADS2 (2 vs1; *P* < .001) and CHA2DS2‐VASc scores (4 vs 2; *P* < .001) were significantly higher in the DM group as all these parameters are integral parts of the score.

**Table 1:** Clinical characteristics of patients

Variable	Diabetic group (n = 122)	Nondiabetic group (n = 120)	*P*‐value
Patients' characteristics			
Age (years), mean ± SD	66.5 ± 7.6	64.4 ± 7.6	.074
Women, n (%)	51 (41.8)	43 (35.8)	.3
BMI, mean ± SD	31.3 ± 5.1	29.3 ± 4.8	.2
BSA (m^2^), mean ± SD	1.97 ± 0.21	1.97 ± 0.2	.8
Comorbidities			
Hypertension, n (%)	102 (83.6)	97 (80.1)	.07
SBP (mmHg), mean ± SD	131.9 ± 22.6	126.3 ± 22.8	.055
DBP (mmHg), mean ± SD	75.9 ± 12.4	74.2 ± 12.5	.3
Dyslipidemia, n (%)	83 (68)	72 (60)	.1
Chronic heart failure, n (%)	6(4.7)	2 (1.7)	.7
Ischemic heart disease, n (%)	32 (26.2)	24 (20)	.2
History of stroke or TIA, n (%)	15 (12.3)	11 (9.2)	.4
CHADS2 score, median (IQR)	2 (2; 3)	1 (0; 1)	<.001
CHA2DS2‐VASc score, median (IQR)	4 (3; 5)	2 (1; 3)	<.001

Abbreviations: BMI, body mass index; BSA, body surface area; DBP, diastolic blood pressure; IQR, interquartile range (25th; 75th percentiles); SBP, systolic blood pressure; TIA, transient ischemic attack.

**Figure 2:**
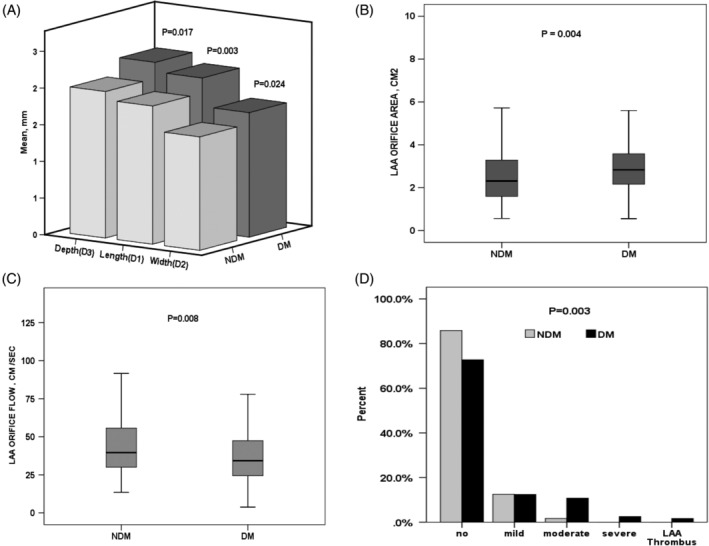
Difference in left atrium appendage characteristic in the diabetic group and the nondiabetic group. A, Depth, length, and width. B, LAA orifice area. C, LAA flow velocity. D, Spontaneous echo contrast distribution. CX, circumflex; D1, length; D2, width; D3, depth; DM, diabetes mellitus; LA, left atrium; LAA, left atrium appendage; non‐DM, nondiabetes mellitus

Echocardiographic and LAA characteristics (Figures 1 and 2 and Table 2) show no significant differences in left ventricular end diastolic diameter, left ventricular end systolic diameter, right ventricular end diastolic diameter, left ventricular ejection fraction, aortic root diameter, ascending aortic diameter, right atrium (RA), and LA area between the two groups. On the other hand, LA‐AP diameter (42.5 ± 6.6 vs 39.6 ± 6.8 mmHg; *P* = .001) and pulmonary artery pressure (32.2 ± 10.1 vs 28.7 ± 7.1 mmHg; *P* = .007) were significantly higher in the DM group than in the non‐DM group. Statistically significant differences were observed in the interventricular septum (11 ± 1.8 vs 10.3 ± 1.5; *P* < .001), although values for both groups are within normal ranges. Compared to the non‐DM group, the DM group had larger LAA diameters, D1‐length (2.09 ± 0.50 vs 1.88 ± 0.54 cm, *P =* .003), D2‐width (1.70 ± 0.48 vs 1.55 ± 0.55 cm, *P =* .024), D3‐depth (2.21 ± 0.75 vs 1.99 ± 0.65 cm, *P* = .017, Figure 2A), LAA orifice area (2.8 ± 1.35 vs 2.3 ± 1.49 cm^2^, *P =* .004, Figure 2B), and LAA number of lobes (2 [2; 3] vs 2 [1; 2]; *P* = .001). In contrast, LAA orifice flow velocity was significantly lower in the DM group than in the non‐DM group (37.3 ± 17.6 vs 43.7 ± 19.5 cm/s; *P* = .008, Figure 2C). The DM group was characterized by a higher rate of SEC (*P =* .003, Figure 2D) and LAA circumference (6.3 ± 1.5 vs 5.9 ± 1.6, *P =* .019, Figures S1 and 2). In multivariate regression analysis of the entire cohort, hypertension (OR = 3.7; 95% CI 1.7‐8.2), DM (OR = 3.1; 95% CI 1.02‐9.8), and D2‐width (OR = 1.8; 95% CI 1.05‐3.2) were independent predictors of LAA flow velocity in the LAA (Table 3).

**Table 2:** Patients' echocardiographic characteristics

Variable	Diabetic group (n = 122)	Nondiabetic group (n = 120)	*P*‐value
Standard echocardiographic measurements			
LVEDD (mm), mean ± SD	50.1 ± 5.7	49.9 ± 5.7	.8
LVESD (mm), mean ± SD	34.4 ± 7.4	34.4 ± 6.1	.3
IVS (mm), mean ± SD	11 ± 1.8	10.3 ± 1.5	.001
LVEF (%), mean ± SD	56.2 ± 8.9	58.5 ± 4.2	.07
RVEDD (mm), mean ± SD	38.8 ± 5.1	38.4 ± 4.8	.5
Aortic root (mm), mean ± SD	31.8 ± 4.1	31.5 ± 3.4	.6
Ascending aorta (mm), mean ± SD	33.3 ± 3.8	32.9 ± 3.9	.4
LA‐AP (mm), mean ± SD	42.5 ± 6.6	39.6 ± 6.8	.001
LA Area (mm), mean ± SD	24.7 ± 5.1	23.2 ± 5.2	.027
RA Area (mm), mean ± SD	19.8 ± 4.8	19.1 ± 4.7	.3
Pulmonary artery pressure (mm Hg), mean ± SD	32.2 ± 10.1	28.7 ± 7.1	.007
E/E′	11.1 ± 4.6	9.7 ± 2.8	.23
E/A ratio	1.2 ± 0.5	1.2 ± 0.3	.97
LAA measurements			
LAA length (cm) D1, median (IQR)	2.09 (1.7; 2.4)	1.88 (1.5; 2.2)	.003
LAA width (cm) D2, median (IQR)	1.70 (1.4; 2)	1.55 (1.2; 1.7)	.024
LAA depth (cm) D3, median (IQR)	2.21 (1.8; 2.6)	1.99 (1.5; 2.3)	.017
LAA area (cm2), median (IQR)	2.8 (2.2; 3.6)	2.3 (1.6; 3.3)	.004
LAA orifice flow velocity (cm/sec), mean ± SD	37.3 ± 17.6	43.7 ± 19.5	.008
LAA number of lobes, median (IQR)	2 (2; 3)	2 (1; 2)	.001
LAA circumference (cm), mean ± SD	6.3 ± 1.5	5.9 ± 1.6	.019
SEC			
No contrast, n (%)	88 (72.7)	103 (85.8)	.003
Mild, n (%)	15 (12.4)	15 (12.5)	
Moderate, n (%)	13 (10.7)	2 (1.7)	
Severe, n (%)	3 (2.5)	0 (0)	
LAA thrombus	2 (1.7)	0 (0)	

Abbreviations: IQR, interquartile range (25th; 75th percentiles); LAA, left atrium appendage; LVEDD, left ventricle end diastolic diameter; LVESD, left ventricle end systolic diameter; IVS, interventricular septum; LVEF, left ventricle ejection fraction; RVEDD, right ventricle end diastolic diameter; LA‐AP, left atrial anterior‐posterior diameter; LA area, left atrial area; RA area, right atrial area; E/A, early to late mitral flow; LAA, left atrium appendage; SEC, spontaneous echo contrast.

**Table 3:** Multiple logistic regression analysis showing independent predictors of slow LAA flow velocity

			95% confidence Interval	
Variable	*P*‐value	Odds ratio	Lower	Upper
Hypertension	.001	3.7	1.7	8.2
Diabetes mellitus	.047	3.1	1.02	9.8
LAA width (D2)	.034	1.8	1.05	3.2
LAA length (D1)	.058	1.3	0.98	2.1
LAA depth (D3)	.065	1.1	0.9	2.5

Abbreviation: LAA, left atrium appendage.

## 4 DISCUSSION

The results of our study demonstrate the presence of LAA differences including LAA orifice enlargement and low LAA flow velocity in DM patients with AFib. In long‐term follow‐up studies, DM was established as an independent risk factor for AFib with atrial structural remodeling as a major culprit of DM‐related AFib.^25^, ^26^ Several potential molecular mechanisms for atrial remodeling leading to AFib in diabetic patients have been implicated including insulin resistance, endothelial dysfunction, collagen deposition, inflammatory response, reduced fibrinolysis, Hb1A1c, renin‐angiotensin‐aldosterone system, and accelerated atherogenesis are associated factors causing structural, electrical, and autonomic heart dysfunction.^11^ Nevertheless, the process is not yet fully understood and studies on LAA geometry have largely been focused on AFib with limited studies on DM patients with AFib.

Diastolic dysfunction is the major contributor of adverse LA/LAA remodeling.^27^, ^28^ LA/LAA enlargement and altered geometry can predispose to stagnation, thrombosis, and subsequent increased risk of thromboembolic stroke.^28^, ^29^ The risk of thrombus formation in the LAA seems to be related to impaired LA and LAA function, reduced contractile function, and elevated filling pressures.^24^, ^30^ In our study, the multivariate logistic regression analysis revealed that hypertension was the main significant risk factor, however, diabetes and LAA width (D2) were also independently and significantly associated factors of slow LAA velocity. Interestingly, the study group patients were predominantly hypertensive patients, indicating that the observed results can mainly be attributed to the DM effects. In addition, the LAA orifice enlargement and reduced orifice flow velocity have already been associated with higher risk of thromboembolism.^31^, ^32^

Currently, 2DTEE is the most widely used and accepted modality to diagnose and exclude the presence of LAA thrombi.^33^, ^34^, ^35^ However, 3DTEE provides better separation and differentiation between adjacent structures, a more complete and comprehensive evaluation of the LAA complex geometry and surrounding structures.^22^, ^23^ The 3DTEE is particularly useful to assess the risk of thromboembolism in multilobe LAA, which is difficult to visualize in its entirety with 2DTEE imaging. The evaluation of LAA with Doppler velocities is important to assess the propensity for LAA thrombus formation.^36^, ^37^ In this study, the 3DTEE imaging revealed different number of LAA lobes with four and five lobes most frequently observed in DM patients, with no significant age‐ or sex‐related differences. In the non‐DM group, two lobes, followed by thee lobes and one lobe, characterized the LAA. In fact, a possible explanation for this observation is that during AFib and ensuing LAA remodeling, some lobes are closed, obstructed, or may open during flow reduction in ischemic coronary events. Shear stress resulting in enlargement of the LA after stiffness of the left ventricle in diabetic patients with diastolic dysfunction can also dilate the LAA and open or close the lobes.^38^ The main limitation of the study is the lack of long‐term follow‐up to evaluate the outcome of DM patients in terms of ischemic stroke or other thromboembolic complications. However, the aim of this study was to evaluate LAA geometric changes in DM patients with AFib, in addition to hypertension and not the prognostic follow‐up.

## 5 CONCLUSIONS

We demonstrated the existence of LAA geometric changes in DM patients with AFib. Adverse LAA remodeling in DM patients have important clinical implications in the prediction of embolic events as well as with decision‐making in terms of optimal prevention and therapy. Consequently, further studies are required to evaluate LAA geometric changes as a contributing factor, alongside hypertension and DM, in thrombus formation and stroke risk in DM patients with AFib. In our study, the morphofunctional modifications of the LAA in DM with Afib may predispose to LAA thrombosis and increased risk thromboembolic events. Consequently, the anatomical and mechanical structural changes in the LAA may become an important additional factor to the traditional CHA_2_DS_2_‐VASc score for an appropriate preventive stroke management in DM patients with AFib.

## CONFLICT OF INTEREST

The authors declare that there is no conflict of interest.

## Supporting information


**Figure S1**
