# Supplementary figures and images for: Difference in left atrial appendage remodeling between diabetic and nondiabetic patients with atrial fibrillation
Source: Clin Cardiol. 2019 Nov 22;43(1):71–7. doi: 10.1002/clc.23292 (PMC6954381; doi:10.1002/clc.23292)

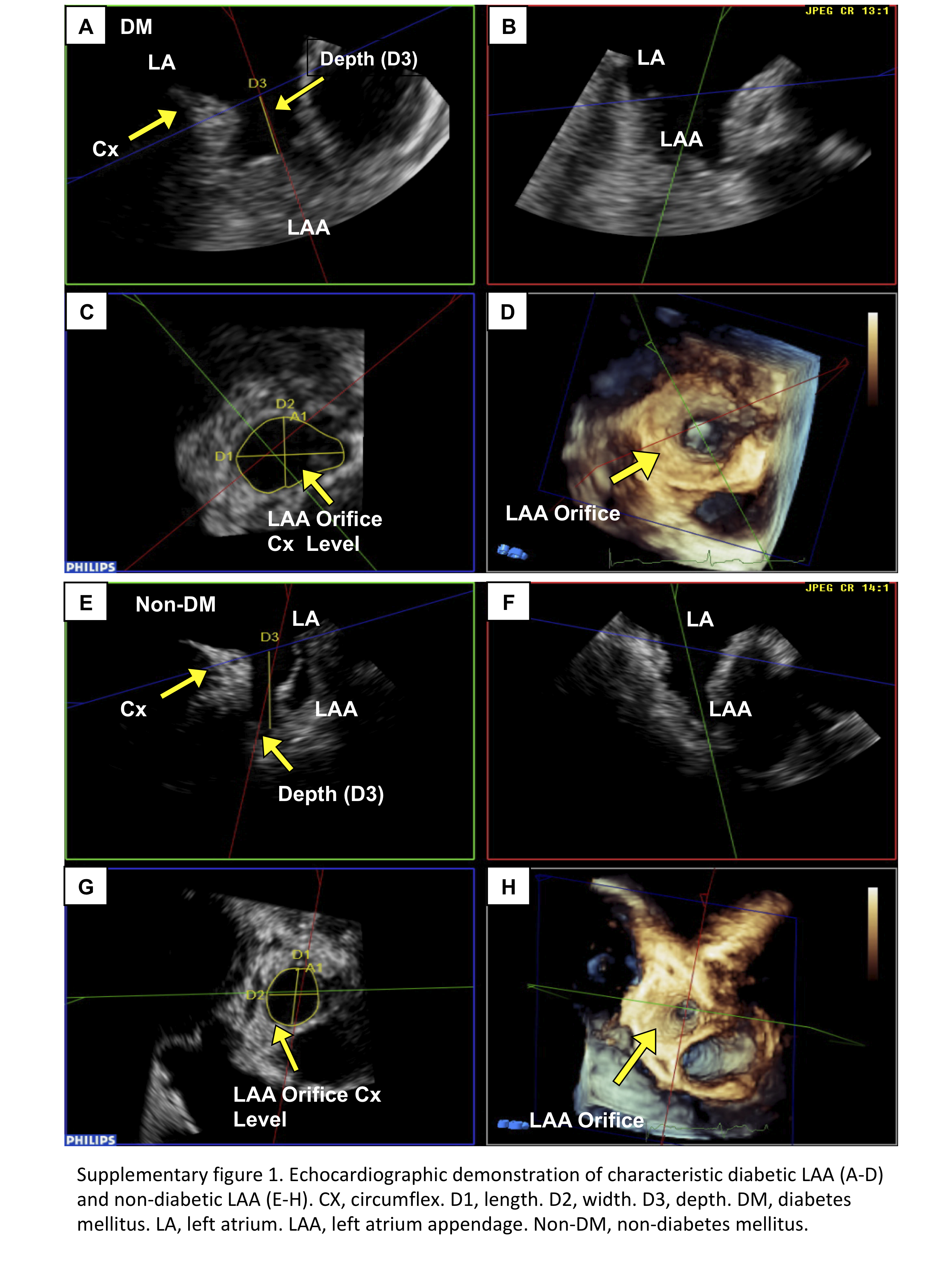

Supplement: Supplementary file 1 — Figure S1 [file CLC-43-71-s001.png]
